# Saskatoon Berry *Amelanchier alnifolia* Regulates Glucose Metabolism and Improves Cardiovascular and Liver Signs of Diet-Induced Metabolic Syndrome in Rats

**DOI:** 10.3390/nu12040931

**Published:** 2020-03-27

**Authors:** Ryan du Preez, Stephen Wanyonyi, Peter Mouatt, Sunil K. Panchal, Lindsay Brown

**Affiliations:** 1 Functional Foods Research Group, University of Southern Queensland, Toowoomba, QLD 4350, Australia; r.dupreez@cqu.edu.au (R.d.P.); Stephen.Wanyonyi@usq.edu.au (S.W.); S.Panchal@westernsydney.edu.au (S.K.P.); 2 School of Health and Wellbeing, University of Southern Queensland, Toowoomba, QLD 4350, Australia; 3 Southern Cross Plant Science, Southern Cross University, Lismore, NSW 2480, Australia; Peter.Mouatt@scu.edu.au

**Keywords:** metabolic syndrome, *Amelanchier alnifolia*, Saskatoon berry, obesity, inflammation, flavonoid, anthocyanin

## Abstract

Saskatoon berry (*Amelanchier alnifolia*) is a potential functional food containing anthocyanins and flavonols, as well as ellagitannins and phenolic acids. We have determined the potential therapeutic effects of Saskatoon berry in diet-induced metabolic syndrome. Nine- to ten-week-old male Wistar rats were randomly assigned to four groups. Two groups were fed on control diets, either corn starch (C) or high-carbohydrate, high-fat diet (H) respectively, for 16 weeks. Two further groups were fed on C or H diet for 16 weeks with Saskatoon berry powder added to the diet for the final 8 weeks (CSSK, HSSK). After 16 weeks, H rats showed symptoms of metabolic syndrome, including increased body weight, visceral adiposity, systolic blood pressure, cardiac fibrosis, plasma concentrations of triglycerides and non-esterified fatty acids, and plasma activities of alanine transaminase and aspartate transaminase. Saskatoon berry intervention normalised body weight and adiposity, improved glucose tolerance, decreased systolic blood pressure, improved heart and liver structure and function with decreased infiltration of inflammatory cells, and decreased plasma total cholesterol. Further, Saskatoon berry normalised liver expression of hexokinase 1 and glycogen phosphorylase and increased glucose 6-phosphatase relative to H rats. These results suggest that Saskatoon berry regulates glycolysis, gluconeogenesis and glycogenesis to improve metabolic syndrome.

## 1. Introduction

Saskatoon berry, *Amelanchier alnifolia,* is a fruit crop native to Canada that is adaptable to extreme cold conditions, high and low altitudes and a range of soil types [1]. Saskatoon berry is consumed as a raw fruit or used as a natural additive to pies, jellies, jams and syrups [1]. Saskatoon berry has found unique applications as a flavouring agent, decreasing the bitter and astringent attributes of natural remedies such as rooibos tea/vitamin D_3_ supplements and thereby enhancing acceptability of flavour [2]. In common with other bright-coloured berries, Saskatoon berry contains polyphenols including flavonoids (anthocyanins, flavonols and flavanols), condensed tannins (proanthocyanidins), hydrolysable tannins (ellagitannins and gallotannins), phenolic acids (hydroxybenzoic and hydroxycinnamic acids, chlorogenic acids), stilbenoids and lignans [3,4,5,6]. Phytochemical concentrations varied according to species, genotype, and growing and post-harvesting conditions [7]. In particular, Saskatoon berry contained anthocyanins such as cyanidin 3-galactoside, cyanidin 3-glucoside and other cyanidin glycosides [8] as well as delphinidin 3-glucoside, malvidin 3-glucoside and malvidin 3-galactoside at higher concentrations than in other berries [9].

Metabolic syndrome is a combination of obesity, hypertension, glucose intolerance or diabetes, fatty liver disease and systemic inflammation that increases the risk of cardiovascular and metabolic diseases [10]. Many foods have been claimed to be effective in the prevention or treatment of the signs of metabolic syndrome [11]. We have established a rat model using a diet high in simple sugars such as fructose as well as saturated and *trans* fats, which results in development of the clinical signs of metabolic syndrome, cardiovascular remodelling and fatty liver [12]. Using this model, we have reported that anthocyanins, especially cyanidin 3-glucoside from purple carrots [13], Queen Garnet plums [14], chokeberries and purple maize [15], attenuated or prevented the infiltration of inflammatory cells in the heart and liver, associated with improved organ function. Furthermore, flavonols such as rutin [16] and quercetin [17] and phenolic acids such as chlorogenic acid [18], as well as ellagitannins [19], were effective on most diet-induced symptoms in this model. Saskatoon berry contains anthocyanidins such as cyanidin glycosides and flavonols such as rutin, as well as ellagitannins and phenolic acids, and this combination should be effective against the signs of metabolic syndrome. In diet-induced-obese mice, Saskatoon berry postponed the increase in body weight, attenuated diet-induced metabolic disorders and vascular inflammation, and lowered the Firmicutes/Bacteroidetes ratio in the gut microbiome [20]. In particular, this could be important to fragile indigenous communities where diabetes and cardiometabolic risk factors remain very common, such as Canadian First Nations and Métis populations [21], living where Saskatoon berry is native or farmed.

The rationale for investigating natural remedies such as Saskatoon berry is the increasing morbidity and mortality associated with metabolic syndrome [22,23]. However, despite the presence of bioactive compounds and some internet claims for the health benefits of Saskatoon berry, there is limited evidence that Saskatoon berry improves health as a functional food in humans with metabolic syndrome. In this study, we investigated the potential of Saskatoon berry to reverse signs of metabolic syndrome as measured by cardiovascular, liver and metabolic parameters and examined changes in glucose metabolism in rats fed a high-carbohydrate, high-fat diet. Furthermore, we measured the transcript abundance of key enzymes in glucose and fatty acid metabolism in order to determine the pathways in energy metabolism that may be modulated by treatment with Saskatoon berry.

## 2. Materials and Methods

### 2.1. Rats and Diets

All experimental protocols were approved by the Animal Ethics Committee of the University of Southern Queensland with approval number 16REA005 under the guidelines of the National Health and Medical Research Council of Australia. Male Wistar rats (9–10 weeks old; 330 ± 2 g; *n* = 48) were purchased from the Animal Resource Centre, Murdoch, WA, Australia and were allowed one week to acclimatise to the new environment before commencing the protocol. Rats were housed in individual cages in a temperature-controlled (21 ± 2 °C) room with an automated 12-h light/dark cycle environment and had free access to food and water. Measurements of body weights and intakes of food and water were performed daily at the start of the light cycle. Rats were randomly divided into four experimental groups, each consisting of 12 rats. Two groups were fed on control diets, either corn starch (C) or high-carbohydrate, high-fat diet (H), respectively, for the entire duration of the protocol (16 weeks). A third group of rats was fed on C diet for the 16 weeks of the protocol with Saskatoon berry powder added to the diet for the final 8 weeks (CSSK). The fourth group of rats was fed on H diet for the 16 weeks of the protocol and with Saskatoon berry powder added to the diet for the final 8 weeks (HSSK). Saskatoon berry was added to the diets as a powder at 26.83 g/kg of food. This concentration of Saskatoon berry in the food was chosen to give an approximate dose of cyanidin glucoside of 8 mg/kg/day in the CSSK rats for comparison with our previous studies on other fruits and vegetables containing cyanidin glucoside [14,15].

Diets were prepared as earlier described [12]. Briefly, the C diet consisted of 570 g corn starch (Agri Food Ingredients, Fitzroy North, VIC, Australia), 155 g powdered rat food (Specialty Feeds, Glen Forrest, WA, Australia), 25 g Hubble, Mendel and Wakeman salt mixture (MP Biomedicals, Seven Hills, NSW, Australia) and 250 mL tap water per kilogram of food. The H diet consisted of 175 g fructose (Tate & Lyle, Wacol, QLD, Australia), 395 g condensed milk (Coles Supermarkets, Melbourne, VIC, Australia), 200 g beef tallow (Carey Brothers Meats, Warwick, QLD, Australia), 155 g powdered rat food, 25 g Hubble, Mendel and Wakeman salt mixture and 50 mL water. C and CSSK rats were given tap water whereas H and HSSK rats were given tap water containing 25% fructose (*w*/*v*). The energy densities of C and H diets were 11.23 kJ/g and 17.83 kJ/g, respectively, and an additional 3.85 kJ/mL in the drinking water for the H diet-fed groups (H and HSSK rats) [12].

### 2.2. Phytochemical Characterisation of Saskatoon Berry Powder

Phytochemical analysis of freeze-dried Saskatoon berry powder was performed using British and American Pharmacopoeia protocols (2016 versions). Dried powders were analysed on an Agilent 1100 Series High Performance Liquid Chromatography (HPLC) System (Agilent Technologies Australia, Mulgrave, VIC, Australia). All solvents and reagents used were HPLC or analytical grade. Anthocyanin content was analysed using a Phenomenex Luna C18 HPLC column (250 × 4.6 mm) as described in the BP2016 monograph for analysis of anthocyanin content in bilberry extracts. The mobile phases were solvent A (8.5% formic acid, Milli-Q water) and solvent B (8.5% formic acid, 22.5% acetonitrile (Scharlau; Chem-Supply, Gilman, SA, Australia), 22.5% methanol, 41.5% water). The gradient started at 7% solvent B, which was increased to 25% over 35 min, then to 65% solvent B over 10 min, at a flow rate of 1 mL/min and an injection volume of 10 µL. Specific detection and calibration for each compound was performed at 535 nm.

References and test samples were prepared in methanol containing 2% hydrochloric acid and 0.1% phosphoric acid. Calibration standards of cyanidin 3-chloride (10 mg) and a standardised bilberry extract (3.34% cyanidin 3-glucoside) (125 mg) were prepared in acidified methanol (25 mL), then diluted in 0.1% phosphoric acid, 2 mL into 100 mL and 5 mL into 20 mL respectively. Saskatoon berry powder (234.3 mg) was extracted in acidic methanol (25 mL), sonicated for 15 min then centrifuged. A 2.5 mL aliquot of the supernatant was then diluted 4-fold with dilute phosphoric acid, equilibrated for 15 min and an aliquot added to a HPLC vial for analysis. Total anthocyanins were calculated as cyanidin 3-glucoside and expressed as mg/100 g.

For flavonoid content, chromatography was performed using a Phenomenex Luna C18 HPLC column (250 × 4.6 mm) with a gradient method as described in the USP2016 *Ginkgo biloba* extract monograph for analysis and limit of rutin and quercetin. The mobile phases were solvent A (0.1% formic acid, Milli-Q water) and solvent B (acetonitrile (Scharlau)) over 45 min. The gradient started at 10% solvent B which was increased to 36% solvent B over 40 min, then to 100% solvent B over 5 min, followed by 5 min washout and return to initial conditions over 10 min, with a flow rate of 1 mL/min. Specific detection and calibration for each compound was performed at 254 nm. Reference standards of rutin and quercetin were prepared in methanol at 0.59 mg/mL and 0.142 mg/mL, respectively, then diluted for a five-point calibration curve. Saskatoon berry powder (261.1 mg) was extracted in methanol (25 mL), sonicated for 15 min then centrifuged. An aliquot (2.5 mL) of the supernatant was then added into an HPLC vial for analysis. The quantity of flavonoids was calculated based on calibration curve of reference standards, peak area at 254 nm and sample dilution. Total flavonoid glycosides were calculated as rutin, quercetin was calculated as quercetin, and results were expressed as mg/100 g.

Analysis of the hydroxycinnamic acid derivatives was also performed using a Phenomenex Luna C18 HPLC column (250 × 4.6 mm), using a gradient method as described in the BP2016 *Echinacea purpurea* monograph method for hydroxycinnamic acid quantification. The mobile phases were solvent A (1% *v*/*v* phosphoric acid, Milli-Q water) and solvent B (acetonitrile (Scharlau)) over 20 min. The gradient started at 10% solvent B which was increased to 22% and 40% solvent B over 13 and then 20 min, followed by 5 min washout and return to initial conditions over 10 min, with a flow rate of 1.5 mL/min. Specific detection and calibration for each compound was performed at 330 nm. Reference standards of chlorogenic acid (Sigma-Aldrich Australia, Castle Hill, NSW, Australia) were prepared in 70% ethanol and diluted for a five-point calibration curve. Saskatoon berry powder (366.0 mg) was extracted in 70% ethanol (25 mL), sonicated for 15 min then centrifuged. An aliquot was then added into a HPLC vial for analysis. The quantity of hydroxycinnamic acids was calculated as chlorogenic acid for total hydroxycinnamic acids and results were expressed as mg/100 g.

### 2.3. Measurements on Live Rats

Systolic blood pressure was measured at 0, 8 and 16 weeks under light sedation by intraperitoneal injection with Zoletil (tiletamine 10 mg/kg, zolazepam 10 mg/kg; Virbac, Peakhurst, NSW, Australia). Measurements were performed using an MLT1010 Piezo-Electric Pulse Transducer (ADInstruments, Bella Vista, NSW, Australia) and an inflatable tail-cuff connected to an MLT844 Physiological Pressure Transducer (ADInstruments) connected to a PowerLab data acquisition unit (ADInstruments) [12].

Oral glucose tolerance tests were performed at 0, 8 and 16 weeks on rats after overnight (12 h) food deprivation. During this time, fructose-supplemented drinking water in H and HSSK rats was replaced with tap water. Basal blood glucose concentrations were determined in tail vein blood using Medisense Precision Q.I.D. glucometer (Abbott Laboratories, Bedford, MA, USA) and glucose test strips (Freestyle Optium Blood Glucose Test Strips, Abbott Diabetes Care Ltd., Witney, Oxon, UK). The rats were given 2 g/kg body weight of glucose as a 40% (*w*/*v*) aqueous glucose solution via oral gavage. Tail vein blood samples were taken at 30, 60, 90 and 120 min following glucose administration [12].

Dual-energy X-ray absorptiometry (DXA) was performed on all rats after 8 and 16 weeks of feeding using a Norland XR46 DXA scanner (Norland Corp., Fort Atkinson, WI, USA). Rats were sedated using intraperitoneal injection of Zoletil (tiletamine 10 mg/kg and zolazepam 10 mg/kg; Virbac). Scans were analysed using the manufacturer’s recommended software for use in laboratory animals (Small Subject Analysis Software, version 2.5.3/1.3.1; Norland Corp.). The precision error of lean mass for replicate measurements, with repositioning, was 3.2% [12]. Visceral adiposity index (%) was calculated as earlier reported [12].

Whole body metabolism was measured at 16 weeks using a four-chamber OxyMax system (Columbus Instruments, Columbus, OH, USA), placing one rat per chamber. Rats had free access to food and water during the experiment. Carbon dioxide production (VCO_2_) and oxygen consumption (VO_2_) were determined from each chamber. Respiratory exchange ratio (VCO_2_/VO_2_) was quantified by OxyMax software (v. 4.86). Energy expenditure was quantified based on the exchange of oxygen for carbon dioxide that occurs during metabolism of food [24].

### 2.4. Measurements on Isolated Organs and Tissues

Terminal euthanasia was induced in all rats via intraperitoneal injection of Lethabarb (pentobarbitone sodium, 100 mg/kg; Virbac), and approximately 6 mL blood was immediately drawn from the abdominal aorta and processed for plasma [12]. Hearts (*n* = 10) were separated into right ventricle and left ventricle with septum for weighing. Livers and abdominal fat pads (retroperitoneal, epididymal and omental) were isolated and weighed (*n* = 10). Organ weights were normalised to the tibial length and the final organ weight is presented in mg of tissue/mm of tibial length [12].

Rapid tissue fixation was undertaken for the final two rats of each group to ensure intact tissues for analysis. Tissues were also collected for histology from two other rats in each group. Part of the heart, liver, small intestine and large intestine from these rats of each group was collected and fixed in 10% neutral buffered formalin for three days. Standard histological procedures were followed to process tissues for staining with haematoxylin and eosin or picrosirius red. Two slides were prepared per tissue specimen and two random, non-overlapping fields per slide were taken to avoid biased analysis. In order to examine collagen distribution in the heart, the tissue was stained with picrosirius red stain and imaged using EVOS FLC microscope (Tokyo, Japan). Small and large intestine sections were stained with haematoxylin and eosin to identify inflammatory cells [12].

Plasma collected during terminal experiments was used to measure enzyme activities and concentrations of biochemical markers. Plasma activities of alanine transaminase and aspartate transaminase, and plasma concentrations of total cholesterol and triglycerides were determined at the School of Veterinary Sciences, The University of Queensland, Gatton, QLD, using kits and controls supplied by Olympus (Tokyo, Japan) on an AU 400 Olympus Analyzer [12]. Non-esterified fatty acids were determined using a commercial kit (Wako Diagnostics, Osaka, Japan) [12] at the School of Veterinary Sciences, The University of Queensland, Gatton, QLD.

Liver samples were taken immediately after euthanasia and completely immersed in RNAlater^®^ (Sigma-Aldrich Australia) in 2 mL microcentrifuge tubes for storage at −80 °C until required. The tissue was thawed on ice and RNAlater drained off before purification of RNA from approximately 100 mg of tissue using the FavorPrep™ Tissue Total RNA Mini Kit (Favorgen, Ping-Tung, Taiwan). The quality and concentration of RNA was determined using the Agilent 2100 Bioanalyzer by loading 1 µL of the RNA preparation onto an Agilent RNA 6000 Nano chip alongside an RNA ladder. Only RNA samples with an RNA integrity number (RIN) greater than nine were used for gene expression analysis. First-strand cDNA synthesis was performed using the SensiFAST cDNA Synthesis Kit (Bioline, Alexandria, NSW, Australia) starting with 1 µg of RNA. Quantitative PCRs were performed using the SsoFast EvaGreen (BioRad, Gladesville, NSW, Australia) method and pre-optimised proprietary Rank 1 KiCqStart primers (catalogue number KSPQ12012G) (Sigma-Aldrich Australia). Transcript quantification was performed using the CFX Manager software (BioRad). β-actin was used as an internal control for PCR. Amplification comprised enzyme activation at 95 °C for 30 s followed by 40 cycles of 95 °C for 30 s, 65 °C for 30 s and melt curve performed from 55 °C to 95 °C in increments of 0.5 °C every 45 s. To determine the effect of Saskatoon berry on glucose metabolism, hexokinase-1, glucose 6-phosphatase, phosphofructokinase 1, glycogen synthase 2 and glycogen phosphorylase transcripts were quantified. The transcripts for acetyl CoA carboxylase, carnitine palmitoyltransferase 1 and peroxisome proliferator-activated receptor α were measured to determine the effects on lipid metabolism. The transcripts for AMP-activated protein kinase were measured to determine the overall effect on energy metabolism. To visualise differences in gene expression between treatments, a 1.2% Agarose (TAE) gel was loaded with the entire 20 µL PCR product after a 40-cycle PCR.

### 2.5. Statistical Analysis

All data are presented as mean ± standard error of the mean (SEM). Results were tested for variance using Bartlett’s test and variables that were not normally distributed were transformed using a log10 function prior to statistical analyses. Data from the four groups were compared by two-way analysis of variance to calculate *p* values for the effects of diet, treatment and the interaction between diet and treatment. When the interactions and/or the main effects were significant, means were compared using the Newman–Keuls multiple comparison post hoc test. A *p* value of <0.05 was considered as statistically significant. All statistical analyses were performed using Prism version 5.00 for Windows (GraphPad Software, San Diego, CA, USA).

## 3. Results

### 3.1. Composition of A. alnifolia Powder

The total flavonoid concentration was 294.13 mg/100 g of berry powder, consisting of 211.79 and 82.34 mg/100 g of quercetin equivalents and rutin, respectively. The total anthocyanin content, calculated as cyanidin 3-glucoside, was 281 mg/100 g of Saskatoon berry powder. The total phenolic acid content based on a chlorogenic acid standard was determined to be 108 mg/100 g of Saskatoon berry powder.

### 3.2. Metabolic, Cardiovascular, Liver and Gastrointestinal Tract Parameters

**Figure 1:**
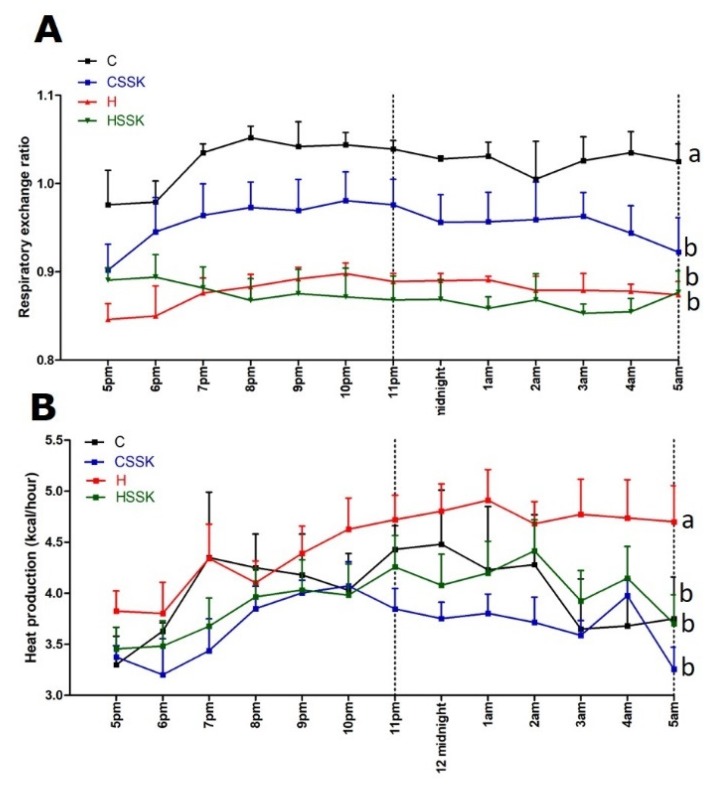
(**A**) Twelve-hour indirect calorimeter data for respiratory exchange ratio and (**B**) heat production. End-point means with unlike superscripts differ (a or b), *p* < 0.05. C: corn starch diet-fed rats; CSSK: corn starch diet-fed rats supplemented with Saskatoon berry powder; H: high-carbohydrate, high-fat diet-fed rats; HSSK: high-carbohydrate, high-fat diet-fed rats supplemented with Saskatoon berry powder.

**Figure 2:**
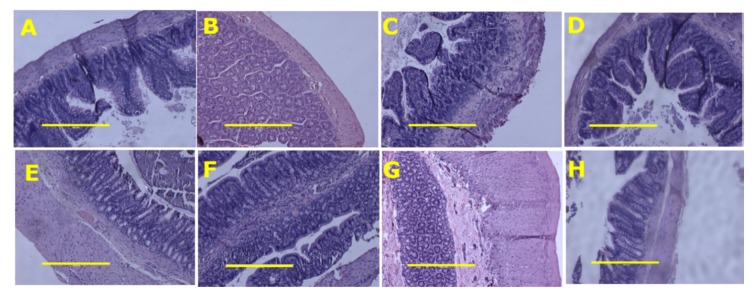
Ileum (top row) and colon (bottom row) structure using haematoxylin and eosin stain in corn starch diet-fed rats (**A**,**E**), corn starch diet-fed rats supplemented with Saskatoon berry powder (**B**,**F**), high-carbohydrate, high-fat diet-fed rats (**C**,**G**) and high-carbohydrate, high-fat diet-fed rats supplemented with Saskatoon berry powder (**D**,**H**). The yellow scale bar is 100 µm (10×).

Food intake was higher in C rats relative to H rats, but energy intake was lower. Food and energy intakes were lower in HSSK rats than in H rats (Table 1). The average anthocyanin, quercetin, rutin and chlorogenic acid intakes were less in HSSK rats than in CSSK rats, as HSSK rats were heavier and ate less (Table 1). There was no difference in water intake between groups during the 8 weeks of treatment. The body weight and feed efficiency were decreased in C, CSSK and HSSK rats compared to H-fed rats (Table 1). Similarly, abdominal circumference, visceral adiposity index, the individual fat pads including retroperitoneal, omental and epidydimal fat, and total abdominal fat were decreased in C, CSSK and HSSK rats compared to H rats (Table 2). Bone mineral density and bone mineral content were higher in H rats than in C rats. There was no difference in bone mineral content and density between HSSK and H rats. There was no difference in lean mass between treatment groups. The respiratory exchange ratio (Figure 1A) was higher in C rats compared to CSSK, H and HSSK rats. Heat production (Figure 1B) was higher in H rats compared to C, CCSK and HSSK rats. Brown fat increased in the following order: C, HSKK, H, CSSK (Table 2). Histology of ileum and colon did not show any structural abnormalities in the treatment groups with normal crypt depth, villi length and goblet cells, and less infiltration of inflammatory cells relative to C rats for CSSK rats and to H rats for HSSK rats (Figure 2).

Basal blood glucose concentrations were higher in H and HSSK rats than in C and CSSK rats (Table 2). The oral glucose tolerance test area under the curve (AUC) was decreased in HSSK compared to H rats and was higher in both than in C rats. CSSK rats had the lowest AUC. The plasma concentrations of triglycerides and non-esterified fatty acids were elevated in H rats compared to C rats. There was no difference in the plasma concentrations of triglycerides and non-esterified fatty acids between HSSK and H rats and, similarly, no difference between CSSK rats and C rats. However, the plasma concentration of total cholesterol was decreased in HSSK and CSSK rats compared to H rats and C rats (Table 2).

**Figure 3:**
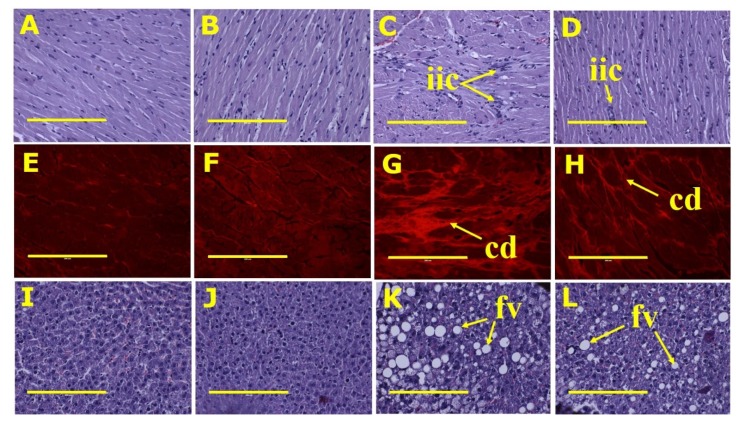
Heart histology: infiltrated inflammatory cells (top row—“iic”) using haematoxylin and eosin stain; collagen deposition (middle row—“cd”) using picrosirius red stain. Liver histology: fat vacuoles (bottom row—“fv”) using haematoxylin and eosin stain in corn starch diet-fed rats (**A**,**E**,**I**); corn starch diet-fed rats supplemented with Saskatoon berry powder (**B**,**F**,**J**); high-carbohydrate, high-fat diet-fed rats (**C**,**G**,**K**); and high-carbohydrate, high-fat diet-fed rats supplemented with Saskatoon berry powder (**D**,**H**,**L**). The yellow scale bar is 200 µm (20×).

Systolic blood pressure and diastolic stiffness constant were higher in H rats than in C rats (Table 2). Both cardiovascular parameters were improved in HSSK rats relative to H rats. Left ventricular + septum wet weights were higher in H rats compared to C rats; CSSK rats were the same as C rats, and HSSK rats were similar to C rats (Table 1). Right ventricular wet weights were not different between groups (Table 1). Histological sections of the left ventricle showed increased infiltration of inflammatory cells and collagen deposition in H rats relative to C rats (Figure 3). HSSK diet decreased infiltration of inflammatory cells and collagen deposition in the left ventricle (Figure 3).

The wet weight of the liver was higher in H rats compared to C rats (Table 2). HSSK rats had decreased liver weight compared to H rats but there was no difference between CSSK and C rats. The plasma activities of aspartate transaminase were elevated in H-fed rats relative to C-fed rats. However, there was no difference in plasma activities of aspartate transaminase between HSSK and H rats nor, similarly, between CSSK and C rats. Compared to C, CSSK and HSSK rats, the plasma activities of alanine transaminase were higher in H rats (Table 2). The HSSK diet normalised plasma alanine transaminase activity. Histological sections (Figure 3) of the inner lobe of the liver revealed elevated counts and size of fat globules in hepatic tissue of H rats. HSSK liver sections showed a decrease in the size of fat globules compared to H rats (Table 2). There were no observable abnormalities in the liver sections of C and CSSK rats.

### 3.3. Gene Expression

**Figure 4:**
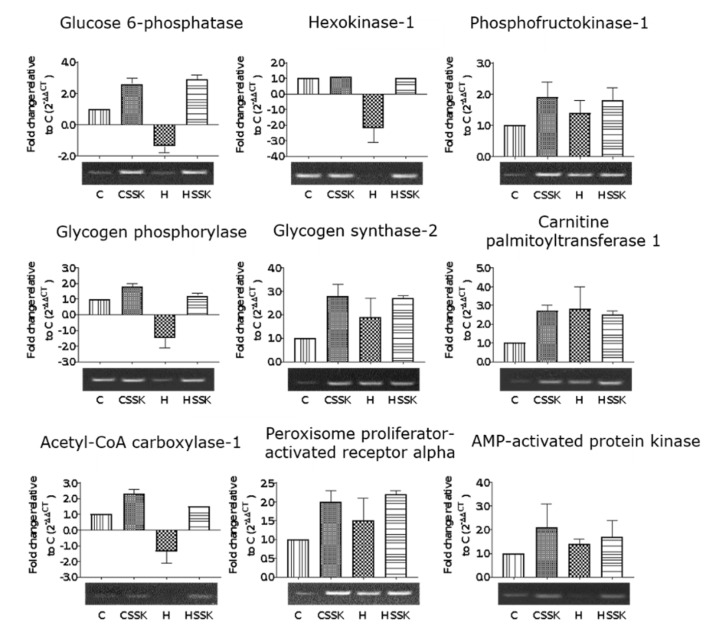
Saskatoon berry supplementation on gene expression of enzymes and transcription factors involved in liver glucose and lipid metabolism. The fold change relative to C was derived from technical duplicates of liver cDNA from three rats per treatment group. Error bars are presented as standard deviations. Changes in gene expression were considered significant if the *p* value was <0.05.

Gene expression for glucose 6-phosphatase, hexokinase 1 and glycogen phosphorylase was decreased in H rats compared to C, CSSK and HSSK rats (Figure 4). There was no difference in the expression of these genes between HSSK and C rats. The expression of phosphofructokinase-1, glycogen synthase 2, peroxisome proliferator-activated receptor α and AMP-activated protein kinase was not different between CSSK, H and HSSK rats. Carnitine palmitoyltransferase-1 expression was increased in CSSK, H and HSSK compared to C rats. There was no difference in the expression of AMP-activated protein kinase between treatment groups. Although samples were screened for peroxisome proliferator-activated receptor α, sterol regulatory element-binding protein 1 and cAMP responsive element binding protein 1, which play important roles in lipid metabolism, the data was variable between individual rats and is not presented.

## 4. Discussion

Functional foods provide nutrition and also prevent or reverse disease states. This study strongly indicates that Saskatoon berry may be an effective functional food for metabolic syndrome. The usefulness of fruits and vegetables as effective functional foods in metabolic syndrome has often been reported, but this concept remains intuitive and possible rather than proven [11]. In this study, we have used our established Wistar rat model of metabolic syndrome, in which an increased intake of simple carbohydrates and saturated and *trans* fats induced signs of metabolic syndrome including increased systolic blood pressure, cardiac fibrosis, cardiac stiffness, abdominal adiposity, fat deposition in the liver, plasma liver enzymes and inflammatory cell infiltration as well as impaired glucose tolerance [13]. We have now demonstrated the potential therapeutic actions of Saskatoon berry, *A. alnifolia*, as a functional food, which improved cardiac and liver structure and function, decreased visceral adiposity, increased the respiratory exchange ratio and improved glucose tolerance. We suggest that anthocyanins such as cyanidin 3-glucoside are the major bioactive compounds in Saskatoon berry, as the doses were similar to in our earlier studies on cyanidin-containing fruits and vegetables in the same model of metabolic syndrome [13,14,15] while the doses of quercetin, rutin and chlorogenic acid were much lower than the effective doses in our earlier studies [16,17,18]. However, low doses of rutin, quercetin and chlorogenic acid may have produced small additive effects to those of the anthocyanins.

Berries are likely to confer their therapeutic effects through their content of anthocyanins, flavonoids, phenolic acids, stilbenoids and lignans [2,4]. These bioactive compounds may work together to reduce obesity, as with the related compounds in rosella (*Hibiscus sabdariffa*) [25]. Anthocyanins improve cardiovascular health, as shown by epidemiological studies leading to clinical studies showing improved surrogate markers of cardiovascular health, such as hypertension, lipid profiles and endothelial function with increased effects in overweight individuals [26]. Thus, anthocyanins are important in the potential prevention and reversal of metabolic syndrome, including obesity and inflammation [27,28]. These clinical studies have been supported by animal studies, including our reports in the same model as this study on purple carrots, Queen Garnet plums, purple maize and chokeberries [13,14,15]. Possible mechanisms include suppression of inflammatory responses, modulation of gene expression (including for antioxidant defences), cell signalling and mRNA expression [26]. Flavonoids also modulate obesity by mechanisms including regulation of food intake, nutrition absorption, adipogenesis, adipocyte lifecycle, thermogenesis, energy consumption and gut microbiota [29]. Flavonoids improve endothelial function, improve peripheral and cerebral blood flow and reduce blood pressure in humans [29], possibly through modulation of the nitric oxide system [30] and bi-directional relationships with the gut microbiota [31]. Phenolic acids such as chlorogenic acid may regulate glucose and lipid metabolism in cardiovascular disease, diabetes, liver steatosis and obesity [32]. Using the same model as this study, we have reported that rutin, quercetin, chlorogenic acid and ellagitannins can modulate the signs of metabolic syndrome [16,17,18,19]. Lignans and stilbenoids such as resveratrol may be useful adjuvants for the treatment of obesity and inflammation, possibly also by influencing the gut microbiota [33]. Thus, novel fruits such as Saskatoon berry which contain these compounds are viable options to be developed as functional foods for metabolic syndrome. Further, Saskatoon berry may act as an antioxidant when added to other foods, thus increasing concentrations of other bioactive ingredients [34].

Few studies have reported responses to Saskatoon berry in the pathophysiological changes that occur in metabolic syndrome. Treatment with 5% Saskatoon berry for 4 weeks decreased markers of endoplasmic stress in the hearts and aortae of wild type and *db*/*db* mice, suggesting potential in attenuating insulin resistance and diabetic cardiovascular complications [35]. This effect was attributed to the high content of cyanidin 3-glucoside and cyanidin 3-galactoside. In rats fed a high-fat, high-sucrose diet with 5% Saskatoon berry powder for 15 weeks, there was a reduced Firmicutes to Bacteroidetes ratio relative to the high-fat, high-sucrose control rats [20]. The fruit peel from Saskatoon berry was abundant in polyphenol compounds and showed the highest anti-oxidative activity [36]. Additionally, Saskatoon berry had anti-microbial, anti-hyperglycaemic and anti-obesity properties.

Systemic therapeutic effects of anthocyanins as in metabolic syndrome rely on bioavailability [37]. Although plasma concentrations of cyanidin glycosides following oral intake are low, the total urinary recovery of anthocyanins and metabolites suggests reasonable oral bioavailability [38]. Further, three consecutive Saskatoon berry supplements 4 h apart in humans showed increased plasma concentrations of some cyanidin and peonidin glycosides [39], suggesting that taking Saskatoon berry with each meal would provide improved health benefits.

A possible mechanism for the improved metabolic response with Saskatoon berry could be improved glucose regulation. In the liver, Akt regulates glucose metabolism by promoting the conversion of glucose to glucose 6-phosphate by stimulating the expression of hexokinase [40]. Akt further increases the translocation of GLUT1 to the plasma membrane and stimulates glycogen synthesis by phosphorylating glycogen synthase kinase 3, thereby indirectly stimulating glycogen synthase 2 activity [40,41]. Additionally, Akt activates glycolysis enzymes indirectly, via hypoxia-inducible transcription factors and downstream activation of phosphofructokinase-1 [42]. There is also evidence suggesting that Akt may ubiquitously regulate catabolic pathways by acting as a negative regulator of AMP-activated protein kinase [43]. It would therefore appear that anthocyanin-rich foods partly improve metabolic parameters by promoting glucose clearance from the bloodstream, especially into storage organs such as the liver.

In this study, supplementation with Saskatoon berry normalised the expression of hexokinase 1, an enzyme that phosphorylates glucose and other hexoses for downstream utilisation in glycolysis and glycogenesis. The phosphorylation of glucose by hexokinase leads to a decrease in intracellular glucose, thereby maintaining a concentration gradient that favours the facilitated transport of glucose into the cells [44,45,46,47]. The influx of glucose leads to increased concentrations of glucose 6-phosphate and a consequent increase in the activity of glucose-metabolic enzymes such as glycogen synthase 2 and phosphofructokinase-1 [47]. Therefore, the absence of normalisation in the expression of glycogen synthase 2 observed in this study would suggest that glycogen synthase 2 may have been regulated post-transcriptionally or even post-translationally. Alternatively, rather than stimulate the uptake and storage of glucose in the form of glycogen, Saskatoon berry stimulated catabolic pathways such as glycolysis and glycogenolysis. This is evident in the normalisation of the expression of glycogen phosphorylase, the enzyme that catalyses the rate-limiting step in glycogenolysis.

Apart from normalising hexokinase 1 expression, Saskatoon berry increased the expression of glucose 6-phosphatase, suggesting a concurrent increase in glycolysis and gluconeogenesis. Under normal metabolic conditions, gluconeogenesis and glycolysis are regulated by reciprocal allosteric controls so that the stimulation of one leads to the inhibition of the other [48]. Conventionally opposing metabolic reactions can be upregulated simultaneously in what is known as futile cycles [49,50,51,52] as in the case of fructose metabolism [53]. Dietary fructose appears to stimulate both glycolysis and gluconeogenesis [54]. However, the differential increase in glucose 6-phosphatase in Saskatoon berry-treated rats relative to H rats could not be attributed to fructose, since H and HSSK diets had identical content of fructose. Measurement of enzyme activity is necessary to resolve this difference in glucose 6-phosphatase gene expression. Similarly, the apparent lack of effect of Saskatoon berry on fatty acid metabolism genes despite the effect on adiposity could be resolved by strategies other than measuring gene expression.

Metabolic syndrome is especially prevalent in indigenous communities. As an example, in 158 youths aged 5–17 years in the Torres Strait islands in northern Queensland, Australia, 38% had higher waist circumference, 27% were hypertensive and 56% had elevated serum insulin concentrations [55]. Further, metabolic syndrome in childhood in an Australian Aboriginal population was associated with subclinical atherosclerosis in 19-year-olds, possibly mediated by increased inflammation [56]. Canadian First Nations populations show an increased incidence of obesity [57] and chronic kidney disease and associated cardiovascular comorbidities [58]. The Canadian government has provided a food subsidy to bring perishable fruits and vegetables to remote rural areas. This approach, together with the support for using traditional foods, may underlie the lower incidence of childhood obesity in First Nations communities than in Alaskan Native communities [59]. Our results suggest that these high prevalence values in Indigenous children and adults could be reduced by increased intake of locally grown functional foods, which may include edible berries with Saskatoon berry as one example.

## 5. Conclusions

Saskatoon berry supplementation in high-carbohydrate, high-fat diet-fed male Wistar rats attenuated metabolic syndrome, notably decreasing cardiac inflammation and collagen deposition and hepatic lipid accumulation. The therapeutic effects for metabolic syndrome are likely to have been mediated by anthocyanins such as cyanidin glycosides, possibly by increased glucose utilisation and especially through glycolysis.

## Figures and Tables

**Table 1:** Physiological responses to Saskatoon berry.

Physiological Variables	C	CSSK	H	HSSK	*p* Value		
					Diet	Treatment	Interaction
8 week body weight, g	361 ± 4 ^b^	369 ± 5 ^b^	457 ± 14 ^a^	437 ± 6 ^a^	<0.0001	0.43	0.07
16 week body weight, g	390 ± 9 ^c^	396 ± 6 ^c^	577 ± 20 ^a^	500 ± 11 ^b^	0.007	0.0001	0.002
9–16 week body weight gain, %	7.4 ± 1.8 ^c^	7.1 ± 1.0 ^c^	18.2 ± 3.1 ^a^	14.5 ± 1.6 ^b^	<0.0001	0.33	0.41
16 week systolic blood pressure, mmHg	111.9 ± 1.8 ^bc^	122.5 ± 0.7 ^b^	141.0 ± 2.6 ^a^	126.4 ± 3.1 ^b^	<0.0001	0.38	<0.0001
Diastolic stiffness constant (κ)	18.2 ± 1.3 ^bc^	21.3 ± 1.0 ^b^	26.9 ± 1.0 ^a^	21.6 ± 0.8 ^b^	0.0001	0.30	0.0003
Left ventricle + septum, mg/mm	20.8 ± 0.6 ^c^	21.0 ± 0.6 ^c^	25.3 ± 0.8 ^a^	22.6 ± 0.7 ^bc^	0.0001	0.08	0.042
Right ventricle, mg/mm	4.0 ± 0.2	4.2 ± 0.6	4.6 ± 0.4	4.6 ± 0.5	0.28	0.83	0.83
Water intake 0–8 weeks, mL/day	38.8 ± 2.5 ^b^	44.4 ± 3.6 ^a^	32.8 ± 1.4 ^c^	34.7 ± 1.0 ^c^	0.002	0.12	0.44
Water intake 9–16 weeks, mL/day	30.8 ± 2.6	34.5 ± 3.0	33.0 ± 2.0	32.0 ± 1.4	0.95	0.57	0.32
Food intake 0–8 weeks, g/day	47.7 ± 1.3 ^a^	48.2 ± 1.2 ^a^	33.6 ± 0.9	32.7 ± 1.4	<0.0001	0.87	0.57
Food intake 9–16 weeks, g/day	43.7 ± 1.1 ^a^	40.5 ± 1.2 ^a^	32.8 ± 1.0 ^b^	27.7 ± 1.3 ^bc^	<0.0001	0.0008	0.42
Saskatoon berry powder intake, g/day	—	1.3 ± 0.04	—	0.9 ± 0.04			
Cyanidin 3-glucoside intake, mg/kg/day	—	7.43 ± 0.21	—	5.18 ± 0.22			
Quercetin intake, mg/kg/day	—	5.61 ± 0.12	—	3.90 ± 0.10			
Rutin intake, mg/kg/day	—	2.18 ± 0.05	—	1.52 ± 0.06			
Chlorogenic acid intake, mg/kg/day	—	2.41 ± 0.06	—	1.68 ± 0.05			
Energy intake 0–8 weeks, kJ/day	534 ± 15 ^b^	540 ± 13 ^b^	728 ± 16 ^a^	701 ± 26 ^a^	<0.0001	0.57	0.37
Energy intake 9–16 weeks, kJ/day	493 ± 13 ^c^	453 ± 13 ^d^	712 ± 19 ^a^	606 ± 22 ^b^	<0.0001	0.0001	0.06
Feed efficiency 0–8 weeks, g/kJ	0.05 ± 0.01 ^c^	0.06 ± 0.01 ^c^	0.19 ± 0.01 ^a^	0.15 ± 0.01 ^b^	<0.0001	0.14	0.016
Feed efficiency 9–16 weeks, g/kJ	0.05 ± 0.01 ^b^	0.06 ± 0.01 ^b^	0.12 ± 0.02 ^a^	0.10 ± 0.01 ^a^	<0.0001	0.62	0.14

Values are presented as mean ± SEM, *n* = 10–12. All groups were compared against each other. Means in a row with superscripts without a common letter (a, b, c or d) differ significantly, if a mean has two letters then that mean is not different from means with either of the same two letters, *p* < 0.05. C, corn starch diet-fed rats; CSSK, corn starch diet-fed rats supplemented with Saskatoon berry powder; H, high-carbohydrate, high-fat diet-fed rats; HSSK, high-carbohydrate, high-fat diet-fed rats supplemented with Saskatoon berry powder.

**Table 2:** Metabolic responses to Saskatoon berry.

Metabolic Variables	C	CSSK	H	HSSK	*p* Value		
					Diet	Treatment	Interaction
16 week bone mineral content, g	12.3 ± 0.4 ^b^	11.8 ± 0.3 ^b^	17.6 ± 0.9 ^a^	17.0 ± 0.5 ^a^	0.0001	0.34	0.93
16 week bone mineral density, g/cm^2^	0.182 ± 0.004 ^b^	0.184 ± 0.002 ^b^	0.190 ± 0.003 ^a^	0.194 ± 0.004 ^a^	0.008	0.29	0.86
16 week lean mass, g	307.3 ± 5.9	324.3 ± 10.6	321.8 ± 7.3	304.3 ± 6.6	0.73	0.97	0.033
16 week fat mass, g	65.4 ± 10.0 ^b^	67.4 ± 8.9 ^b^	226.8 ± 25.7 ^a^	188.0 ± 15.7 ^a^	<0.0001	0.27	0.22
8 week abdominal circumference, cm	16.6 ± 0.5 ^b^	18.3 ± 0.1 ^ab^	19.7 ± 0.5 ^a^	20.7 ± 0.1 ^a^	<0.0001	0.0005	0.34
16 week abdominal circumference, cm	17.7 ± 0.4 ^c^	18.8 ± 0.2 ^b^	23.5 ± 0.7 ^a^	21.6 ± 0.2 ^ab^	<0.0001	0.35	0.001
Visceral adiposity, %	5.6 ± 0.5 ^c^	5.3 ± 0.2 ^c^	11.3 ± 0.5 ^a^	8.8 ± 0.4 ^b^	<0.0001	0.002	0.012
Retroperitoneal fat, mg/mm	218 ± 23 ^c^	233 ± 13 ^c^	706 ± 67 ^a^	469 ± 33 ^b^	<0.0001	0.008	0.003
Epididymal fat, mg/mm	116 ± 10 ^c^	74 ± 7 ^d^	324 ± 20 ^a^	191 ± 14 ^b^	<0.0001	<0.0001	0.005
Omental fat, mg/mm	116 ± 17 ^c^	127 ± 9 ^c^	315 ± 19 ^a^	220 ± 19 ^b^	<0.0001	0.015	0.003
Total abdominal fat, mg/mm	451 ± 43 ^c^	433 ± 22 ^c^	1345 ± 97 ^a^	880 ± 57 ^b^	<0.0001	0.0003	0.0007
Brown fat, mg/mm	21.8 ± 2.6 ^c^	33.9 ± 1.9 ^a^	31.4 ± 1.8 ^ab^	29.7 ± 2.1 ^b^	0.21	0.018	0.002
8 week 0 min [blood glucose], mmol/L	2.8 ± 0.2 ^b^	2.9 ± 0.1 ^b^	3.4 ± 0.1 ^a^	3.0 ± 0.1 ^a^	0.011	0.26	0.07
16 week 0 min [blood glucose], mmol/L	3.4 ± 0.2 ^ab^	3.4 ± 0.1 ^ab^	4.2 ± 0.2 ^a^	3.8 ± 0.1 ^a^	0.0004	0.21	0.21
8 week OGTT-AUC, mmol/L × min	647 ± 25 ^d^	703 ± 21 ^c^	820 ± 21 ^a^	756 ± 12 ^b^	<0.0001	0.85	0.005
16 week OGTT-AUC, mmol/L × min	599 ± 10 ^c^	547 ± 18 ^d^	712 ± 34 ^a^	590 ± 11 ^b^	0.0005	0.0001	0.10
Liver, mg/mm	230 ± 15 ^c^	233 ± 5 ^c^	399 ± 23 ^a^	326 ± 12 ^b^	<0.0001	0.026	0.016
Liver fat vacuoles area, fat vacuoles/200 µm^2^	8.6 ± 1.0 ^c^	9.2 ± 1.3 ^c^	98.1 ± 8.8 ^a^	74.3 ± 5.9 ^b^	<0.0001	0.051	0.042
Plasma aspartate transaminase, U/L	108.5 ± 10.6 ^b^	118.1 ± 8.4 ^b^	169.7 ± 22.2 ^a^	137.3 ± 14.3 ^ab^	0.022	0.50	0.22
Plasma alanine transaminase, U/L	39.8 ± 4.6 ^b^	45.3 ± 4.6 ^a^	54.8 ± 5.4 ^a^	38.7 ± 5.2 ^b^	0.42	0.32	0.044
Plasma triglycerides, mmol/L	0.57 ± 0.07 ^b^	0.65 ± 0.10 ^b^	1.86 ± 0.22 ^a^	2.25 ± 0.27 ^a^	<0.0001	0.35	0.54
Plasma total cholesterol, mmol/L	1.43 ± 0.08 ^c^	1.42 ± 0.07 ^c^	1.98 ± 0.10 ^a^	1.64 ± 0.07 ^ab^	<0.0001	0.039	0.051
Plasma non-esterified fatty acids, mmol/L	1.72 ± 0.24 ^b^	1.59 ± 0.20 ^bc^	3.44 ± 0.40 ^a^	3.28 ± 0.45 ^a^	<0.0001	0.70	0.97

Values are presented as mean ± SEM, *n* = 10–12. All groups were compared against each other. Means in a row with superscripts without a common letter (a, b, c or d) differ significantly; if a mean has two letters then that mean is not different from means with either of the same two letters, *p* < 0.05. C: corn starch diet-fed rats; CSSK: corn starch diet-fed rats supplemented with Saskatoon berry powder; H: high-carbohydrate, high-fat diet-fed rats; HSSK: high-carbohydrate, high-fat diet-fed rats supplemented with Saskatoon berry powder; OGTT-AUC: oral glucose tolerance test area under the curve.
